# Adipose-Derived Mesenchymal Stem Cells from Ventral Hernia Repair Patients Demonstrate Decreased Vasculogenesis

**DOI:** 10.1155/2014/983715

**Published:** 2014-03-17

**Authors:** Jeffrey Lisiecki, Jacob Rinkinen, Oluwatobi Eboda, Jonathan Peterson, Sara De La Rosa, Shailesh Agarwal, Justin Dimick, Oliver A. Varban, Paul S. Cederna, Stewart C. Wang, Benjamin Levi

**Affiliations:** Department of Surgery, University of Michigan, 1500 E. Medical Center Drive, Ann Arbor, MI 48109, USA

## Abstract

*Introduction.* In adipose tissue healing, angiogenesis is stimulated by adipose-derived stromal stem cells (ASCs). Ventral hernia repair (VHR) patients are at high risk for wound infections. We hypothesize that ASCs from VHR patients are less vasculogenic than ASCs from healthy controls. * Methods.* ASCs were harvested from the subcutaneous fat of patients undergoing VHR by the component separation technique and from matched abdominoplasty patients. RNA and protein were harvested on culture days 0 and 3. Both groups of ASCs were subjected to hypoxic conditions for 12 and 24 hours. RNA was analyzed using qRT-PCR, and protein was used for western blotting. ASCs were also grown in Matrigel under hypoxic conditions and assayed for tubule formation after 24 hours. *Results.* Hernia patient ASCs demonstrated decreased levels of VEGF-A protein and vasculogenic RNA at 3 days of growth in differentiation media. There were also decreases in VEGF-A protein and vasculogenic RNA after growth in hypoxic conditions compared to control ASCs. After 24 hours in hypoxia, VHR ASCs formed fewer tubules in Matrigel than in control patient ASCs. * Conclusion.* ASCs derived from VHR patients appear to express fewer vasculogenic markers and form fewer tubules in Matrigel than ASCs from abdominoplasty patients, suggesting decreased vasculogenic activity.

## 1. Introduction

Ventral hernias are common and morbid complications of abdominal midline laparotomies; once a patient develops such a hernia, they are at risk for recurrent hernia formation via the same wound [1]. The presence of the initial hernia and the high risk of recurrence are indicative of a breakdown in the normal wound healing process. One central component of the wound healing process is the restoration of the metabolic capacity of damaged tissue through angiogenesis and vasculogenesis, two separate but related processes [2]. The restoration of the microvascular network is a complex process that depends on the interaction and coordination of numerous cytokines and growth factors with the extracellular matrix [3]. Deficiencies in any of the essential growth factors for vasculogenesis may disrupt the normal wound healing process; the delivery of exogenous versions of these growth factors is currently a promising concept in the treatment of chronic wounds such as hernias [4].

There also exists a compelling link between surgical wounds and bone formation. For decades, there have been case reports of heterotopic ossification developing in the scars left by abdominal operations [5–7]. More recently, this process has even been observed on the acellular dermal matrices commonly used to repair abdominal wounds [8]. Though this is an uncommon finding and its etiology is yet to be determined, it may be suggestive of the commonality that exists between wound healing and bone formation [9], which includes their dependence on the formation of blood vessels.

Numerous growth factors are responsible for mediating the processes of angiogenesis and vasculogenesis including vascular endothelial growth factor (VEGF); when considering all of the isoforms of VEGF, VEGF-A has been studied most extensively for its role in initiating these processes. VEGF is the growth factor that promotes endothelial cell migration early in the process of angiogenesis [10, 11] and also promotes the proliferation of the endothelial cells that will eventually form blood vessels [12–14]. Logically, a major stimulus for VEGF-A release is hypoxia, or low oxygen tension, which frequently occurs in the wound environment; the resulting angiogenesis restores oxygen tension and metabolic activity to the tissue [15]. Animal models have supported the importance of VEGF-A in restoring the angiogenic capabilities of diabetic limb wounds [16] through an increase in vessel formation [17]. VEGF-A is not the only growth factor involved in revascularization; HIF-1a is also upregulated in the wound healing process, which in turn promotes VEGF production [18].

These vasculogenic cytokines are also crucial to the formation of new blood vessels to support bone formation. VEGF-A, for example, is secreted by mesenchymal stem cells during the process of osteogenesis, and in turn it stimulates bone mineralization [19]. Furthermore, the process of distraction osteogenesis has been shown to be associated with increased expression of factors, including VEGF-A, which promote blood vessel formation [20]. One more recent* in vitro* study demonstrated that coculture of more mature endothelial cells (HUVECs) with less mature osteogenic cells (the study used ASCs and OPECs) increases bone nodule formation [21]. Burn injury, another cause of heterotopic ossification, has been associated with increased vascularization of ASCs [22].

One of the main sources of many of these angiogenic and vasculogenic cytokines is the population of mesenchymal cells present in the adipose tissue. These adipose-derived stem cells (ASCs) have been shown to secrete high levels of VEGF and other angiogenic cytokines. Additionally, ASCs are known to have the ability to participate in tubulization, or the formation of de novo blood vessels in concert with endothelial cells. ASCs, having both osteogenic and vasculogenic potential, are also particularly interesting in the field of tissue engineering, especially with the aim of repairing wounds and regrowing bone, where they may prove to be a good cell source for forming vascular networks [23]. Thus, it is possible that augmentation of the angiogenic capacity of ASCs in midline repairs can enhance or accelerate the healing process [24]. Furthermore, when ASCs are placed in hypoxic conditions, they proliferate and upregulate their production of VEGF, increasing their wound-healing capacities [25]. After a midline laparotomy, we believe that ASCs no longer maintain the same vasculogenic potential that was present prior to their first operation. Specifically, we hypothesize that ASCs harvested from patients with previous failed hernia repair are less vasculogenic than those from patients undergoing elective abdominoplasties.

## 2. Methods

### 2.1. Chemicals and Supplies

Medium, fetal bovine serum, and penicillin/streptomycin were purchased from Gibco Life Technologies, (Carlsbad, CA). Cell culture wares were purchased from Corning, Inc. (San Mateo, CA). Unless otherwise specified, all other chemicals were purchased from Sigma-Aldrich (St. Louis, MO).

### 2.2. Cell Harvest

Human adipose-derived stromal cells were harvested from subcutaneous adipose tissue derived from the abdomens of two patients undergoing ventral hernia repair by components separation, and from three patients undergoing abdominoplasty (to be used as control cells) as previously described [26–32]. Lipoaspirate was digested with a type II collagenase solution at 37°C. Cells were pelleted by means of centrifugation and filtered at 100 *μ*m pore size, and primary cultures were established at 37°C, 5% carbon dioxide, in Dulbecco's modified Eagle medium with 10% fetal bovine serum. As this study took place over several months, a total of five lines were derived.

### 2.3. Cell Proliferation Assays

Human adipose-derived stromal cells were seeded onto six-well plates at a density of 5,000 cells per well. All assays were performed in triplicate wells. Two hernia cell lines and one control cell line were used. Cells were plated with standard growth medium (Dulbecco's modified Eagle medium and 10% fetal bovine serum), 1% penicillin/streptomycin. Cells were lifted and counted using a hemocytometer and light microscope on days 1, 3, 5, and 7.

### 2.4. *In Vitro* Culture Assays

For experiments involving isolation of RNA, human adipose-derived stromal cells were seeded onto six-well plates at a density of 80,000 cells per well as previously described [26–29, 33]. All assays were performed in triplicate wells. After attachment, cells were treated with standard growth medium (Dulbecco's modified Eagle medium and 10% fetal bovine serum), 1% penicillin/streptomycin or osteogenic differentiation medium (Dulbecco's modified Eagle medium, 10% fetal bovine serum, 100 *μ*g/mL ascorbic acid, and 10 mM *β*-glycerophosphate), and 1% penicillin/streptomycin. Cells were maintained for 3 days in osteogenic differentiation medium. For differentiation media RNA studies, one cell line each of hernia and abdominoplasty cells were used. For the hypoxia RNA study, two cell lines each of hernia and abdominoplasty cells were used.

### 2.5. Matrigel Tubule Assay

Matrigel (BD Biosciences, Franklin Lakes, NJ) was thawed and placed in four-well chamber slides at 37°C for 30 minutes to allow solidification. Then, 50,000 stromal cells from either hernia or abdominoplasty patient adipose tissue were plated alone on Matrigel and incubated at 37°C under 1% oxygen for 12 hours. Two hernia cell lines and two abdominoplasty cell lines were used for this assay. Tubule formation was defined as a structure exhibiting a length four times its width. Experiments were performed with *n* = 6. Tubule counts were determined in 10 randomly selected fields per well using an inverted Leica DMIL light microscope (Leica Microsystems GmbH, Wetzlar, Germany) at 100x magnification as described previously [34].

### 2.6. Western Blot Analysis of Vascular Signaling

The endogenous activation of the VEGF signaling pathway in human adipose-derived stromal cells was investigated by immunoblotting analysis of VEGF-A as described previously [35]. One hernia cell line and one control cell line were used for each Western blot analysis. Subconfluent human adipose-derived stromal cells were washed twice with phosphate-buffered saline and starved in serum-free medium overnight. Then, the cells were washed twice with ice-cold phosphate-buffered saline and lysed with cold lysis buffer (50 mM of 4-(2-hydroxyethyl)-1-piperazineethanesulfonic acid, pH 7.5, 150 mM of sodium chloride, 1 mM of ethylenediaminetetraacetic acid, 10% glycerol, 1% Triton-X-100, and 25 mM of sodium fluoride) containing 1 mM of sodium orthovanadate and Protease Inhibitor Cocktail (Sigma-Aldrich). Cell lysates were assayed for protein concentration by bicinchoninic acid assay. Aliquots (50 to 100 g) of cell lysate were electrophoresed on 12% Tris-HCl sodium dodecyl sulfate polyacrylamide gel electrophoresis gels (Precast NuPAGE gels; Invitrogen, Life Technologies, Carlsbad, CA) and transferred onto Immobilon-P membrane (Millipore Corp., Bedford, MA). Antibodies against VEGF-A were used (Abcam). A horseradish peroxidase-conjugated anti-rabbit antibody (1 : 8000) was used as a secondary antibody. Alpha-Tubulin antibody was used to control for equal loading and transfer of the samples. All bands in the immunoblots were normalized with the loading controls (alpha-tubulin) and quantified by densitometry.

### 2.7. Statistical Analysis

Demographic information about the patients was compared using *t*-tests. For the cell proliferation, western blot, and PCR statistics, we computed the mean and standard deviation of multiple data points. For the Matrigel tubule formation assay, we calculated the mean of each individual's count of tubules/HPF and the standard deviation of these counts. A cutoff of *P* < 0.05 has been defined for statistical significance.

## 3. Results

### 3.1. Demographics Were Similar between Our Patient Groups

The average age was not significantly different between the hernia and control groups (36.2 versus 49.9 years, *P* = 0.35). Similarly, average BMI was not significantly different between hernia and control groups (35.7 versus 28.0, *P* = 0.19, Table 1). The hernia patients consisted of one man and one woman. All three of the control patients were female. For the hernia patients, the operation from which we collected fat was the patient's sixth and third abdominal operations; none of these operations were for hernia repair. For the two of the control patients, the operation from which we collected adipose tissue was the patient's first abdominal operation; for one of the control patients, the adipose tissue was collected from their second abdominal operation.

### 3.2. ASCs from Hernia Patients Proliferate More Rapidly Than Those from Abdominoplasty Patients

**Figure 1:**
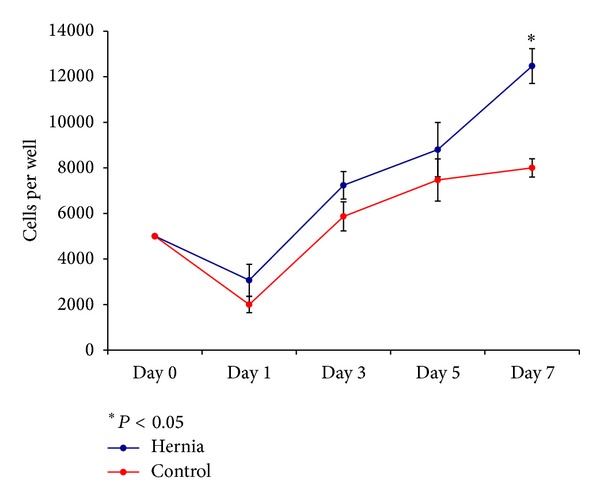
Cell proliferation assay. Average number of cells per well at cell culture days 0, 1, 3, 5, and 7 for hernia and control cell lines. Error bars represent standard deviations. *P < 0.05.

In our cell proliferation studies, we found that hernia patient ASCs proliferated more after seven days compared to our control ASCs (12466.7 ± 764.6 cells per well compared to 8000 ± 400 cells per well, *P* = 0.0064, Figure 1). This difference in rates of proliferation, though it is less pronounced after three days of growth in cell culture, ensures that any decreases in vasculogenic activity that are observed in hernia patient ASCs are a product of decreased vasculogenic potential and not a product of sampling a smaller population of cells.

### 3.3. Hernia Patients Have Decreased VEGF-A Expression by Western Blot, Which Is Further Mitigated by Hypoxic Stress

**Figure 2:**
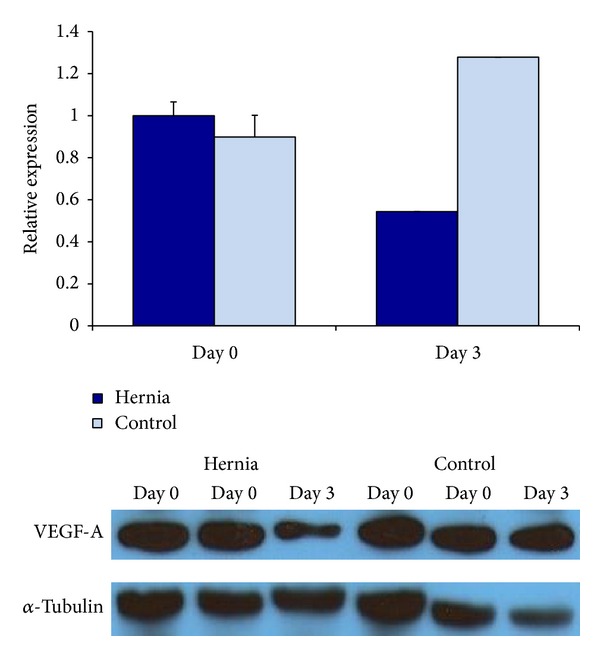
Relative VEGF-A protein expression in hernia and control ASCs by western blotting, after 0 and 3 days of growth in differentiation media. Hernia ASCs in blue and control ASCs in light blue.

**Figure 3:**
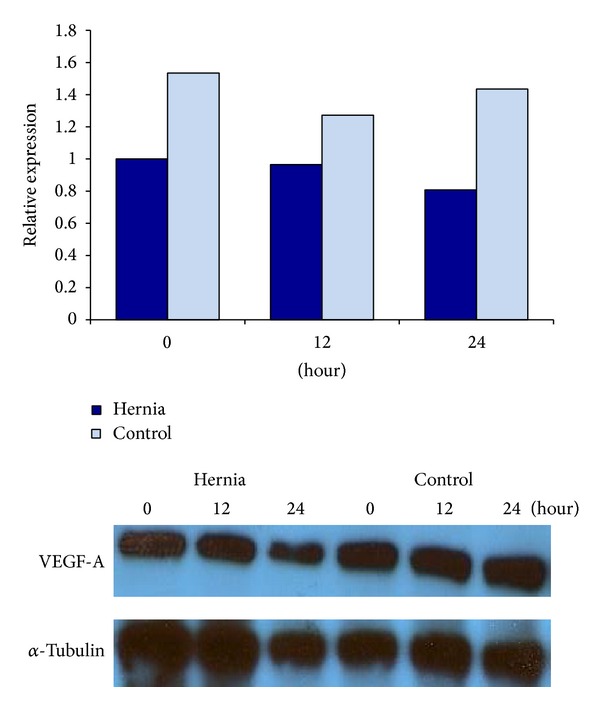
Relative VEGF-A protein expression in hernia and control ASCs by western blotting, after 0, 12, and 24 hours of incubation in hypoxic conditions. Hernia ASCs in blue and control ASCs in light blue.

At baseline, without vasculogenic differentiation, there was no significant difference between hernia and control cells. After 3 days in culture, the hernia patient ASCs demonstrated decreased levels of VEGF-A protein on western blotting relative to control patient ASCs (Figure 2). Unfortunately, due to a limited number of samples, we were unable to assess if this change achieved statistical significance. After incubation in hypoxic conditions, the hernia ASCs demonstrated a decrease in VEGF-A protein expression. The hernia patient ASCs began at a lower baseline level of VEGF-A protein at 0 hours of hypoxia, and the amount of VEGF-A decreased after 12 and 24 hours of incubation in hypoxic conditions. Again, we were unable to assess if this change achieved statistical significance. The control patient ASCs began with a higher level of VEGF-A expression and experienced a lesser decrease in VEGF-A concentrations at the 24-hour time point (Figure 3). Thus, we demonstrate that ASCs from the subcutaneous fat of hernia patients demonstrate decreased VEGF-A production relative to those derived from control patients in the hypoxic conditions.

### 3.4. Hernia Patient ASCs Demonstrate Less Vasculogenic Gene Expression Compared to Control

**Figure 4:**
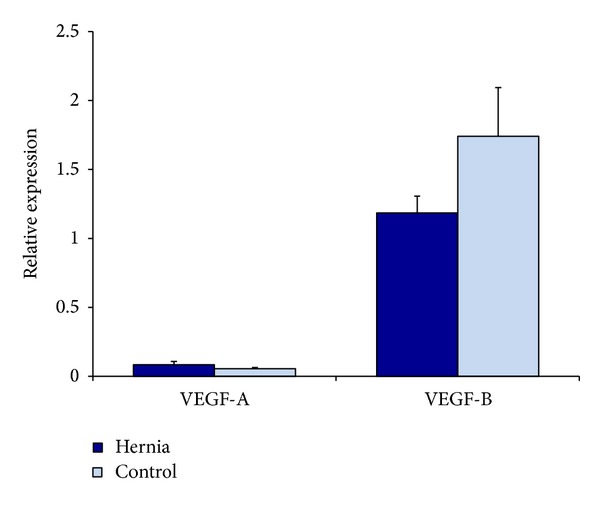
Relative vasculogenic RNA levels in hernia and control ASCs by QRT-PCR, after 3 days of growth in differentiation media. Hernia ASCs in blue and control ASCs in light blue.

**Figure 5:**
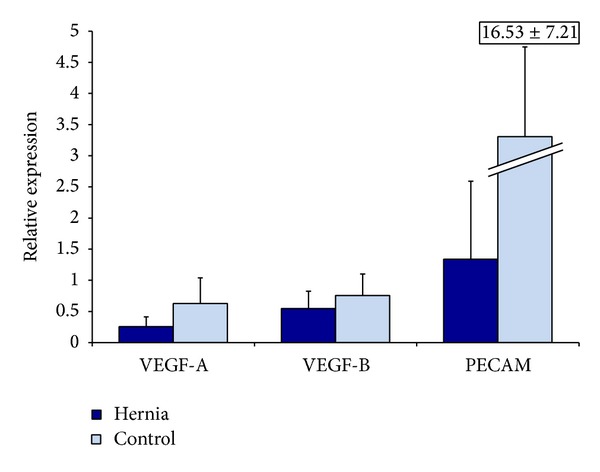
Relative VEGF-A, VEGF-B, VEGF-R, HIF-1A, and PECAM RNA levels in hernia and control ASCs by QRT-PCR, after 12 hours of incubation in hypoxic conditions. Hernia ASCs in blue and control ASCs in light blue.

We next analyzed mRNA expression of our hernia and control cells. We found that hernia patient ASCs demonstrate a decrease in their levels of VEGF-B relative to control after three days of culture (Figure 4). These relative changes, however, failed to achieve statistical significance. After 12 hours of incubation in hypoxic conditions, we observe several key differences between hernia and control patient ASCs. In the hernia ASCs, we observed relative decreases in VEGF-A, VEGF-B, and PECAM after 12 hours of incubation in hypoxic conditions (Figure 5). Thus, we find that hernia ASCs seem to trend toward lower levels of vasculogenic RNA than control ASCs after incubation in hypoxic conditions that simulate wound conditions. This trend, however, does not achieve statistical significance.

### 3.5. Hernia ASCs Demonstrate a Blunted Ability to Undergo Tubulogenesis Compared to Control

**Figure 6:**
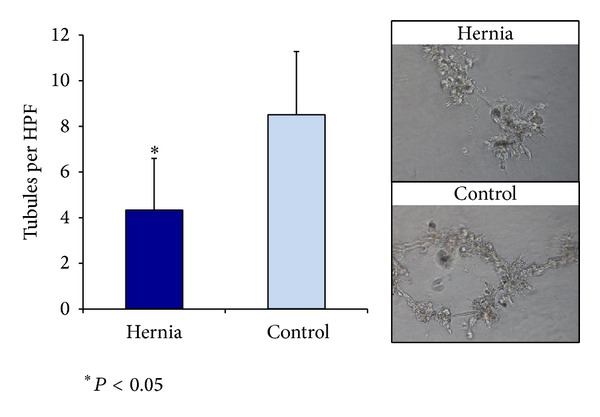
Tubules per HPF formed in Matrigel after incubation in hypoxic conditions (left), with examples of high-powered fields for hernia and control ASCs (right). *P < 0.05.

When we plated hernia and control ASCs in Matrigel and incubated them for 24 hours in hypoxic conditions, we found that the hernia ASCs formed fewer tubules per high-powered field than control patient ASCs (4.33 ± 2.27 tubules/HPF versus 8.51 ± 2.77 tubules/HPF, *P* = 0.0018, Figure 6). Thus, in addition to decreased vasculogenic signaling, hernia derived ASCs demonstrate a decreased ability to form tubules* in vitro*.

## 4. Discussion

Here we demonstrate that ASCs derived from ventral hernia patients demonstrate decreased vasculogenic signaling under normoxic and hypoxic conditions. VEGF-A is an important marker of the initiation of vasculogenesis and angiogenesis and a promoter of endothelialization [10–14]. This suggests that the ASC populations present in the fat of ventral hernia patients are less active in the initial steps of endothelialization than their counterparts in healthy control patients. Analysis of gene expression demonstrates that VEGF-A, VEGF-B, and PECAM are decreased after incubation in hypoxic conditions. Our findings in these cell populations after incubation in hypoxia may be the most pertinent to the conditions of a hernia wound site. The center of healing wounds, such as those resulting from midline laparotomies, is hypoxic, and this hypoxia interferes with the normal angiogenic processes of healing [36]. In response to the hypoxia and metabolic changes that take place in this tissue HIF-1A transcription increases, promoting the accumulation of VEGF [37]. The hernia patient ASCs began with a lower level of VEGF-A protein compared to control patients at 0 hours of hypoxia, and VEGF-A continued to decrease after 12 and 24 hours of incubation in hypoxia, to a greater extent than in control patient ASCs. This suggests that the ASCs from hernia patients inherently produce less VEGF than their counterparts from control patients and that they have a diminished VEGF response to hypoxia. Since hypoxia approximates the low oxygen tension present at a wound site (such as the wound of a ventral hernia), this finding is highly suggestive of an impairment in the ability of this cell population to initiate angiogenesis and vasculogenesis to revascularize wounded tissue. This idea is further supported by our results after culturing these cells on Matrigel in hypoxic conditions. Tubulogenesis on Matrigel approximates the endothelialization and vasculogenesis that takes place with these cells in human extracellular matrix. We found that, after being grown in hypoxic conditions on Matrigel, our hernia patient ASCs formed fewer tubules than control patient ASCs. These individual differences that we have observed in the vasculogenic capacity of ASCs may also explain the uncommon occurrence of heterotopic bone formation in abdominal surgical incisions. It is possible that these individuals are undergoing increased vasculogenesis to repair (or attempt to repair) their abdominal wounds and are concomitantly developing bone in these regions.

The revascularization of wounded tissue is essential both for successful wound healing and for preventing infection of the wound site by maintaining oxygen tension. Collagen deposition in the wound site is proportional to the oxygen tension and perfusion of the site [38]. Wound hypoxia is especially prominent after abdominal operations immediately following the surgery and is not readily visible to clinicians [39]. Several studies have demonstrated that low oxygen tension in wound tissue is correlated with an increased risk of wound site infection [40].

Further studies in this matter will be crucial in determining why patients who suffer from ventral hernias have impaired revascularization of the wound site, that is, the metabolic and genetic differences contributing to the findings we observe in this paper. Animal* in vivo* studies will ultimately be necessary to confirm the impairment of vasculogenesis and angiogenesis in this patient population. In future studies, we also hope to investigate the* in vivo* transplantation of these cells in mouse models to confirm our findings and to better understand the differences in vasculogenesis and tubulogenesis in a model that more closely resembles the human condition. Ultimately, our goal is to find a therapeutic method to increase the vasculogenic capacity of ASCs that could be used perioperatively to improve the healing of the wound site in hernia patients and, accordingly, decrease the risks of dehiscence and infection in these patients.

We recognize that this paper has several shortcomings. Our patient sample size is relatively small, with two experiment patients and three control patients in the study. Furthermore, the hernia patients recruited for the study tended to be more medically complicated than the control patients undergoing abdominoplasty. We hope that future studies with more patients will confirm our findings. We also recognize that component separation is a relatively uncommon procedure for ventral hernia repair. In addition, there are also some patients who undergo this operation do not have sufficient amounts of fat to remove, modify, and reintroduce to enhance wound healing. Furthermore, this study only focuses on the ASCs, which are responsible for the production of provasculogenic signaling molecules as well as some tubulization but are not the primary cells involved in vasculogenesis. Further studies should compare the endothelial cell populations between hernia and control patients to better understand the vasculogenic processes responsible for inadequate wound healing.

## 5. Conclusion

In this paper we demonstrate that adipose-derived stem cells derived from ventral hernia patients demonstrate decreased expression of the vasculogenic cytokines necessary to revascularize wounded tissue, in differentiation media and under hypoxic conditions. Individual differences in the vasculogenic capability of abdominal fat may also help explain the reports of bone formation in abdominal operation wounds. These findings suggest that the mechanism behind recurrent ventral hernias is an impairment in the angiogenic and vasculogenic pathways.

## Figures and Tables

**Table 1:** Demographic information. Average age and body mass index for hernia and control groups, with *P* values for comparison.

	Hernia	Control	*P* value
Age	36.2	49.9	0.35
Body mass index	35.7	28.0	0.19

## References

[1] Read RR, Cameron JL (1995). Ventral, epigastric, umbilical, spigelian and incisional hernias. *Current Surgical Therapy*.

[2] Velazquez OC (2007). Angiogenesis and vasculogenesis: inducing the growth of new blood vessels and wound healing by stimulation of bone marrow-derived progenitor cell mobilization and homing. *Journal of Vascular Surgery*.

[3] Tonneson MG, Feng X, Clark RA (2000). Angiogenesis in wound healing. *Journal of Investigative Dermatology Symposium Proceedings*.

[4] Barrientos S, Stojadinovic O, Golinko MS, Brem H, Tomic-Canic M (2008). Growth factors and cytokines in wound healing. *Wound Repair and Regeneration*.

[5] Eidelman A, Waron M (1973). Heterotopic ossification in abdominal operation scars. *Archives of Surgery*.

[6] Apostolidis NS, Legakis Ch. N, Gregoriadis GC, Androulakakis PA, Romanos AN (1981). Heterotopic bone formation in abdominal operation scars: report of six cases with review of the literature. *The American Journal of Surgery*.

[7] Lehrman A, Pratt JH, Parkhill EM (1962). Heterotopic bone in laparotomy scars. *The American Journal of Surgery*.

[8] Tam V, Zelken J, Sacks JM (2013). Total heterotopic ossification of an acellular dermal matrix used for abdominal wall reconstruction. *BMJ Case Reports*.

[9] Leis VM, Cotlar AM (2003). Fractured heterotopic bone in a midline abdominal wound. *Current Surgery*.

[10] Suzuma K, Takagi H, Otani A, Honda Y (1998). Hypoxia and vascular endothelial growth factor stimulate angiogenic integrin expression in bovine retinal microvascular endothelial cells. *Investigative Ophthalmology & Visual Science*.

[11] Senger DR, Ledbetter SR, Claffey KP, Papadopoulos-Sergiou A, Perruzzi CA, Detmar M (1996). Stimulation of endothelial cell migration by vascular permeability factor/vascular endothelial growth factor through cooperative mechanisms involving the *α*v*β*3 integrin, osteopontin, and thrombin. *The American Journal of Pathology*.

[12] Morbidelli L, Chang C-HO, Douglas JG, Granger HJ, Ledda F, Ziche M (1996). Nitric oxide mediates mitogenic effect of VEGF on coronary venular endothelium. *American Journal of Physiology*.

[13] Pepper MS, Ferrara N, Orci L, Montesano R (1992). Potent synergism between vascular endothelial growth factor and basic fibroblast growth factor in the induction of angiogenesis in vitro. *Biochemical and Biophysical Research Communications*.

[14] Goto F, Goto K, Weindel K, Folkman J (1993). Synergistic effects of vascular endothelial growth factor and basic fibroblast growth factor on the proliferation and cord formation of bovine capillary endothelial cells within collagen gels. *Laboratory Investigation*.

[15] Silver IA (1969). The measurement of oxygen tension in healing tissue. *Progress in Respiratory Research*.

[16] Walder CE, Errett CJ, Bunting S (1996). Vascular endothelial growth factor augments muscle blood flow and function in a rabbit model of chronic hindlimb ischemia. *Journal of Cardiovascular Pharmacology*.

[17] Galiano RD, Tepper OM, Pelo CR (2004). Topical vascular endothelial growth factor accelerates diabetic wound healing through increased angiogenesis and by mobilizing and recruiting bone marrow-derived cells. *The American Journal of Pathology*.

[18] Elson DA, Ryan HE, Snow JW, Johnson R, Arbeit JM (2000). Coordinate up-regulation of hypoxia inducible factor (HIF)-1*α* and HIF-1 target genes during multi-stage epidermal carcinogenesis and wound healing. *Cancer Research*.

[19] Mayer H, Bertram H, Lindenmaier W, Korff T, Weber H, Weich H (2005). Vascular endothelial growth factor (VEGF-A) expression in human mesenchymal stem cells: autocrine and paracrine role on osteoblastic and endothelial differentiation. *Journal of Cellular Biochemistry*.

[20] Pacicca DM, Patel N, Lee C (2003). Expression of angiogenic factors during distraction osteogenesis. *Bone*.

[21] Valenzuela CD, Allori AC, Reformat DD (2013). Characterization of adipose-derived mesenchymal stem cell combinations for vascularized bone engineering. *Tissue Engineering A*.

[22] Peterson JR, de la Rosa S, Sun H (2013). Burn injury enhances bone formation in heterotopic ossification model. *Annals of Surgery*.

[23] Szpalski C, Barbaro M, Sagebin F, Warren SM (2012). Bone tissue engineering: current strategies and techniques—part II: cell types. *Tissue Engineering B*.

[24] Nie C, Yang D, Xu J, Si Z, Jin X, Zhang J (2011). Locally administered adipose-derived stem cells accelerate wound healing through differentiation and vasculogenesis. *Cell Transplantation*.

[25] Lee EY, Xia Y, Kim W-S (2009). Hypoxia-enhanced wound-healing function of adipose-derived stem cells: increase in stem cell proliferation and up-regulation of VEGF and bFGF. *Wound Repair and Regeneration*.

[26] Levi B, James AW, Xu Y, Commons GW, Longaker MT (2010). Divergent modulation of adipose-derived stromal cell differentiation by TGF-*β*1 based on species of derivation. *Plastic and Reconstructive Surgery*.

[27] Levi B, James AW, Wan DC, Glotzbach JP, Commons GW, Longaker MT (2010). Regulation of human adipose-derived stromal cell osteogenic differentiation by insulin-like growth factor-1 and platelet-derived growth factor-*α*. *Plastic and Reconstructive Surgery*.

[28] Levi B, Wan DC, Glotzbach JP (2011). CD105 protein depletion enhances human adipose-derived stromal cell osteogenesis through reduction of transforming growth factor *β*1 (TGF-*β*1) signaling. *The Journal of Biological Chemistry*.

[29] Levi B, Hyun JS, Nelson ER (2011). Nonintegrating knockdown and customized scaffold design enhances human adipose-derived stem cells in skeletal repair. *Stem Cells*.

[30] Levi B, James AW, Glotzbach JP, Wan DC, Commons GW, Longaker MT (2010). Depot-specific variation in the osteogenic and adipogenic potential of human adipose-derived stromal cells. *Plastic and Reconstructive Surgery*.

[31] James AW, Levi B, Nelson ER (2011). Deleterious effects of freezing on osteogenic differentiation of human adipose-derived stromal cells in vitro and in vivo. *Stem Cells and Development*.

[32] James AW, Levi B, Commons GW, Glotzbach J, Longaker MT (2010). Paracrine interaction between adipose-derived stromal cells and cranial suture-derived mesenchymal cells. *Plastic and Reconstructive Surgery*.

[33] Levi B, James AW, Nelson ER (2011). Human adipose-derived stromal cells stimulate autogenous skeletal repair via paracrine hedgehog signaling with calvarial osteoblasts. *Stem Cells and Development*.

[34] Thangarajah H, Vial IN, Chang E (2009). IFATS collection: adipose stromal cells adopt a proangiogenic phenotype under the influence of hypoxia. *Stem Cells*.

[35] Li S, Quarto N, Longaker MT (2010). Activation of FGF signaling mediates proliferative and osteogenic differences between neural crest derived frontal and mesoderm parietal derived bone. *PLoS ONE*.

[36] Knighton DR, Silver IA, Hunt TK (1981). Regulation of wound-healing angiogenesis—effect of oxygen gradients and inspired oxygen concentration. *Surgery*.

[37] Dor Y, Porat R, Keshet E (2001). Vascular endothelial growth factor and vascular adjustments to perturbations in oxygen homeostasis. *American Journal of Physiology*.

[38] Jonsson K, Jensen JA, Goodson WH (1991). Tissue oxygenation, anemia, and perfusion in relation to wound healing in surgical patients. *Annals of Surgery*.

[39] Chang N, Goodson WH, Gottrup F, Hunt TK (1983). Direct measurement of wound and tissue oxygen tension in postoperative patients. *Annals of Surgery*.

[40] Hopf HW, Hunt TK, West JM (1997). Wound tissue oxygen tension predicts the risk of wound infection in surgical patients. *Archives of Surgery*.

